# Vestibular function in children underperforming at school

**DOI:** 10.1016/S1808-8694(15)30141-5

**Published:** 2015-10-18

**Authors:** Eloisa Sartori Franco, Ivone Panhoca

**Affiliations:** a MSc in Speech and Hearing Therapy at PUC - SP. Professor at the Speech and Hearing Therapy Program at Universidade Metodista de Piracicaba - UNIMEP. Audiology Internship Supervisor - UNIMEP; b PhD in Sciences at the Language Studies Institute at UNICAMP. Professor at the School of Speech and Hearing Therapy at PUC - Campinas. External accredited advisor of the ”Child and Adolescent Health” program, Department of Pediatrics, FCM/UNICAMP; c This study was done under the auspices of the Graduate Program on Children and Adolescent Health - Department of Pediatrics - FCM/UNICAMP. Field work was conducted at the School of Health Sciences - FACIS - Speech and Hearing Therapy Program - UNIMEP

**Keywords:** learning, labyrinthine disease, vertigo

## Abstract

Learning is a complex, dynamic process, structured from motor and perception skills which, when cortically processed, give birth to cognition. Balance is a fundamental neurological function that helps us maintain proper postures, an essential factor in learning and a sign or neurologic maturity. **Aim**: this paper aims to study vestibular function in children underperforming at school. **Study design**: this is a cross-sectional study. **Materials and method**: eighty-eight children with ages ranging between 7 and 12 years attending the public schools of Piracicaba from 2004 to 2006 were enrolled. All children were interviewed, submitted to ENT examination, hearing tests, and vestibular examination. **Results**: fifty-one percent of the participants had no reported difficulties at school, whereas 49.0% were underperforming at school. Under vestibular examination, 73.3% of the children performing well at school had normal findings, whereas 32.6% of the underperforming children had normal test results. Unilateral and bilateral irritative peripheral vestibular alterations were found in 67.4% of the underperformers and in 26.7% of the children not experiencing difficulties at school. **Conclusion**: all vestibular alterations found had an irritative peripheral origin. There was a statistically significant association between vestibular alteration and poor performance at school.

## INTRODUCTION

Learning is a complex, dynamic process, structured from motor and perception skills which, when cortically processed, give rise to cognition. Disorders in specific areas of the central nervous system connected to notions of bodily schemes, space, and time, constitute the neuropathologic basis for perceptomotor alterations and sensations disorders that may result in learning impairments^1^.

One-hundred and three children with and without learning disorders were enrolled in this study. Sixty (58.2%) children did not report learning disorders and 43 (41.7%) reported difficulties at school^2^.

Genetic factors, peripheral sensorial disease, neurogenic infirmities, diseases in general, social and cultural handicap, and learning disorders may negatively impact performance at school^3^.

Alterations in basic function integration, bodily schemes, spatial orientation, rhythm, fine motor coordination, laterality, analysis and synthesis functions, integration of parts into a whole, symbolization, language and its many aspects, impulse, attention and memory are neurogenic disorders that may produce learning impairment^4^.

Static balance is an important neurological function for the maintenance of postures adequate for learning. Dynamic balance is also an important evolutional function that provides insights on neurological maturity. Reports in the literature indicate that children with less developed function in this area have increased probability of having learning difficulties^5^.

The vestibular system, the proprioceptors, and the cerebellum are responsible for functions such as muscle tone, posture, balance, eye-motor coordination, and spatial orientation. This system seems to be largely involved in school learning processes^6^.

Narciso et al.^7^ looked at vestibular variations in children and observed that 47% of the subjects had complaints related to performance at school. They found that vestibular disorders may be associated with learning and motor impairments.

According to Campos et al.^8^ infantile vestibular disorder may considerably affect communication skills, psychological status, and performance at school. Low performance at school may also be indicative of labyrinthine disease.

Caovilla et al.^9^ found that early identification of infantile vestibular disease and etiologic treatment are of paramount importance in preventing the complications that frequently involve motor development and language acquisition. Vestibular tests should be done in every child suspected for vestibular disorder, although it is not easy to obtain precise accounts of the symptoms from the parents or the child.

Functional vestibular tests can be done through electronystagmography and vectonystagmography. Digital vectonystagmography is one of the most widely employed tests in our practice to assess vestibular function, as it offers increased diagnostic sensitivity by measuring parameters connected to vestibular-eye-motor function and comparing stimuli to responses, apart from identifying the direction of the phenomena^10^.

Ganança et al.^11^ studied 30 children with poor performance at school and found alterations under computerized nystagmography in 20.0% of the subjects.

According to Caovilla et al.^12^ the tests included in digital vectonystagmography are eye-motor (eye motion, spontaneous and semi-spontaneous nystagmus, saccadic movement, and optokinetic nystagmus) and vestibular tests (rotational and thermal stimulation).

Abnormalities in voluntary saccadic control have been observed in many development disorders such as: dyslexia; learning impairments; attention deficit and hyperactivity disorder^13^.

Eye motion patterns manifested during reading require alternation between saccade and fixation. It starts off with saccade covering 8 to 10 words combined with eye fixation and ends with prolonged saccade to restart reading another line^14^.

The saccadic pathway involves various regions of the cerebral cortex, cerebellum, and brainstem. Parameters such as latency, velocity, and accuracy of saccadic movements can be used to assess how effectively the central nervous system (CNS) manages rapid eye movement. Few CNS disorders remain undetected when latency, velocity, and accuracy of saccadic movement is rigorously measured^10^.

Vestibular alterations found in dyslexic children have led authors Frank and Levinson^15^ to postulate that vestibular disorders and spontaneous nystagmus may interfere with sequential eye fixation required for reading.

Pendulum tracking is another type of eye motion that results from looking at a moving target used to assess the integrity of the eye-motor system in controlling slow eye motion, as they are affected by CNS and vestibular system disorders.

Following the teacher move around in the classroom, copying from the blackboard, reading, writing, and focusing require good eye-motor function and unaffected vestibular interconnections.

Optokinetic nystagmus is a rhythmic involuntary unconscious automatic ocular phenomenon. It may be reproduced when patients are asked to follow dots that move back and forth in opposite directions. It is an exteroceptive response that compensates the movement of the environment through psycho-optical impulses. Optokinetic nystagmus may be altered in CNS syndromes and vestibular disorders^10^.

Horak et al.^16^ analyzed vestibular function in children with and without reading and writing impairment and found alterations in the vestibulo-ocular reflex in 20% of the subjects with poor performance at school, and that 7% of the children without impairments had vestibular alterations.

The rotational stimulation produced in the decreasing rotational pendulum test allows the assessment of labyrinthine compensation status and its directional preponderance^17^.

Ayres^18^ looked at nystagmic movements in post-rotational stimulation in children with learning impairments and saw reduced post-rotational nystagmus in 50% of the children with learning impairment and exacerbated post-rotational nystagmus in 13% of them.

Thermal stimulation done in caloric tests is the most sensitive of conventional vestibular tests done in patients without specific complaints or anomalies in other vestibular assessment steps.

Ganança et al.^11^ studied 30 children with poor performance at school and found alterations under computerized nystagmography in 20.0% of the subjects, all in peripheral topographic diagnosis. According to the authors, most alterations were seen in the caloric test.

Ganança^19^ conducted a study with 64 children affected by language disorders without complaints of dizziness and found increased rates of vestibular alteration in the peripheral topographic diagnosis.

Relevant findings were observed in the studies by Franco and Caetanelli^20^. Computerized vectonystagmography was performed in school-aged children and 20.7% of the subjects were found to have unilateral and bilateral peripheral irritative vestibular disorders.

Sensorial stimulation is fundamental for the development of language in children. When stimulated from an early age, children develop good coordination and focus. They also develop their creativity and self-confidence, and improve the chances of not having learning disorders.

This study aimed at analyzing vestibular function in school-aged children underperforming at school to better understand the triggering vestibular disorders and to consequently allow the development of future studies that address early intervention.

## MATERIALS AND METHOD

### Study type

This experimental study was approved by the Ethics Committee at our institution under permit 423/2003. It was conducted using the facilities and equipment of a clinic belonging to a university located in the countryside of São Paulo.

### Case selection

The sample was taken from a group of children aged between 7 and 12 years enrolled in public schools in the city of Piracicaba seen at the clinic between 2004 and 2006.

Two groups were assessed separately, one made up of children not reporting learning impairment and another containing children underperforming at school. The children were interviewed alongside their parents or caretakers before they were assigned to either of the groups. The following indicators, among others, were considered: inability to acquire new knowledge at the pace classmates do; not following in-class assignments; and information retention record.

One-hundred children accompanied by their parents or caretakers were invited to participate in the study. They were advised of their role in the study in the clinic.

An instructional meeting was carried out to provide participants with information and free informed consent forms. Testes with participants were scheduled after authorization was given by their parents or caretakers. All subjects agreed to participate and to have results divulged as set on Resolution 196/96.

- Inclusion criteria - group underperforming at school: the sample contained children complaining of low grades at school; their tone hearing thresholds between 500Hz and 8000Hz were equal to or under 15 dBHL^21^, ^22^; type A tympanometry and contralateral and ipsilateral acoustic reflexes present bilaterally at 500Hz and 4000Hz^23^.
- Inclusion criteria - group without learning impairment: the sample contained children that did not report any learning impairment; their tone hearing thresholds between 500Hz and 8000Hz were equal to or under 15 dBHL 21, 22; type A tympanometry and contralateral and ipsilateral acoustic reflexes present bilaterally at 500Hz and 4000Hz ^23^.
- Exclusion criteria - both groups: children under 7 and over 12, those reporting symptoms or hearing and visual disorders that could interfere with research findings.

## PROCEDURES

### Interview

All children and parents were interviewed with the purpose of gathering information on complaints of a vestibular origin, with emphasis on the presence or absence or vertigo, exploring associated complaints, mainly those connected to hearing, neurovegetative symptoms, and neurologic cases suspected for involvement of the posterior fossa.

### Otorhinolaryngological examination

Both groups were examined to exclude ear, nose, and throat disorders that could impact the hearing and vestibular systems.

### Audiological examination

Audiological examination consisted of tone threshold audiometry done through air and bone conduction when required, speech recognition tests, speech recognition threshold, and acoustic impedance tests as described by Mangabeira Albernaz et al.^23^. Speech and tone audiometry tests were done in a soundproof booth and a MADSEN MIDIMATE 622 audiometer was used; for impedance tests we used a MADSEN ZO-72 device.

The criteria defined by Glorig and Davis^21^ and Mangabeira Albernaz et al.^22^ were adopted to characterize normal hearing patterns in various age groups and normal hearing thresholds.

Audiological test outcomes were used only as part of the inclusion criteria.

### Vestibular examination

The children submitted to vestibular examination were told to refrain from having coffee, tea, chocolate, and any labyrinth stimulating drug for 72 hours before the tests.

Vestibular tests were done as determined by Caovilla et al.^12^ both in terms of sequence and test interpretation parameters. Vestibular test outcomes were interpreted as defined by Ganança et al.^24^.

Vectonystagmography was performed using a digital computerized vectonystagmograph (VECWIN) with three channels to acquire and record data, an EVR 03 visual stimulator, and an NGR 05 NEUROGRAFF ELETROMEDICINA LTDA ear air calorimeter.

### All subjects were thus submitted to the following

Nystagmus or position vertigo investigation

According to the definitions set forth by Caovilla et al.^12^.

Eye movement biologic calibration

A visual stimulator was used to perform eye movement biologic calibration.

Spontaneous and semi-spontaneous nystagmus investigation

A visual stimulator (light bar) was used to perform spontaneous nystagmus investigation.

Saccadic movement investigation

A visual stimulator (light bar) was used to perform saccadic movement investigation.

Pendulum tracking investigation

A visual stimulator (light bar) was used to perform pendulum tracking investigation.

Optokinetic nystagmus investigation

A visual stimulator (light bar) was used to perform this investigation.

Peri-rotational nystagmus investigation

A YOSHI rotational pendulum chair was used in this test.

Post-caloric nystagmus investigation

An ear air calorimeter was used to investigate post-caloric nystagmus.

Assessment parameters

The analysis was performed as defined by Caovilla et al.^12^, to find data of interest for vestibular function semiology.

### Analysis criteria

Vestibular test interpretation was done according to the parameters defined by Ganança et al.^24^.

### Statistical method

The following tests were applied in the analysis of the vestibular test results, given the nature of the variables being analyzed:

• Parametric: Student”s t-test, controlled by Levene”s test25, to compare the means between two analyzed variables;
• Non-parametric: Mann-Whitney”s test 26 to verify associations between variables.

A confidence interval of 95% was used based on the mean and standard deviation values for the analyzed variables.

For all tests the level of hypothesis rejection and nullity was set at 0.05 or 5% and significant values were highlighted.

Program SPSS (Statistical Package for Social Sciences) release 13.0 was used to perform statistical calculations.

## RESULTS

Table 1 shows the distribution of the sample in relation to gender and school performance.

**Table 1:** Sample distribution in relation to gender and school performance (n=88).

Learning Impairmenta	Gender		
	female	male	Total
	22	23	45
No	48,9%	51,1%	100,0%
	14	29	43
Yes	32,6%	67,4%	100,0%
	36	52	88
Total		59,1%	100,0%

p = 0,121

Table 2 shows the results of Student”s t-test controlled by Lavene”s test for Equality of Variances so as to check the possible differences between the means of parametric variables for eye-motor calibration parameters, namely latency, velocity, and accuracy.

**Table 2:** Sample distribution in relation to eye-motor calibration parameters and school performance (n=88).

Variable	Learning impairment	n	Mean	Standard deviation	Significance (p)
	yes	43	157,00	81,68	
latency_D_1	no	45	147,31	90,25	0,531
	yes	43	160,35	50,32	
velocity_D_1	no	45	151,13	50,42	0,460
	yes	43	85,35	23,86	
accuracy_D_1	no	45	84,99	22,23	0,822
	yes	43	188,36	98,34	
latency_E_1	no	45	169,52	85,17	0,438
	yes	43	143,84	51,52	
velocity_E_1	no	45	151,05	53,64	0,515
	yes	43	87,18	17,95	
accuracy_E_1	no	45	88,03	15,10	0,796

a≤0,05

**Figure 1:**
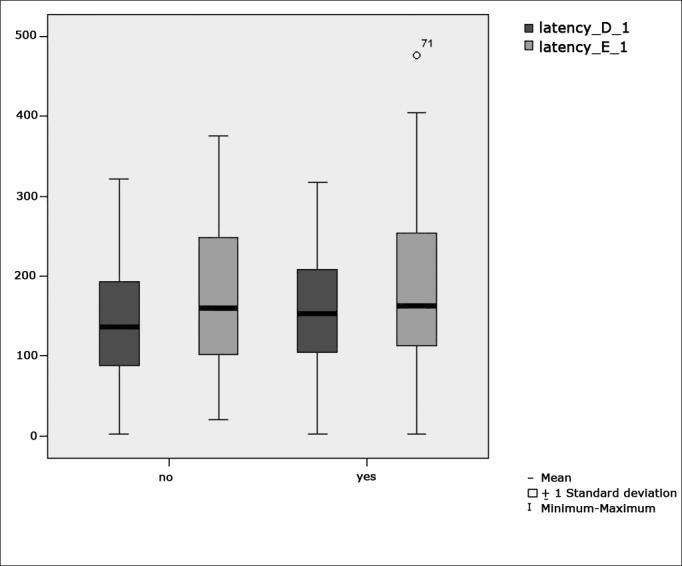
Box plot for right and left calibration eye-motor parameter (latency) per group. (Student”s t-test controlled by Lavene”s test for Equality of Variances between the means of parametric variables of interest).

Figure 1 features a box plot containing the statistical analysis for eye-motor calibration parameter latency.

**Figure 2:**
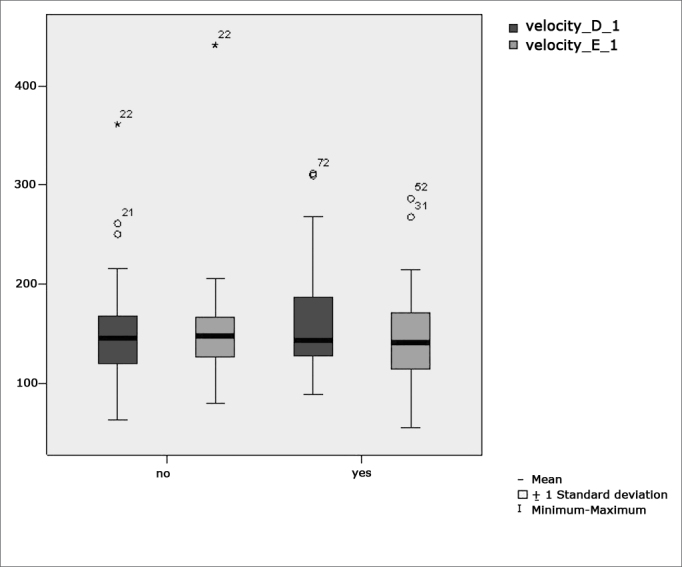
Box plot for right and left calibration eye-motor parameter (velocity) per group. (Student”s t-test controlled by Lavene”s test for Equality of Variances between the means of parametric variables of interest).

Figure 2 features a box plot containing the statistical analysis for eye-motor calibration parameter velocity.

**Figure 3:**
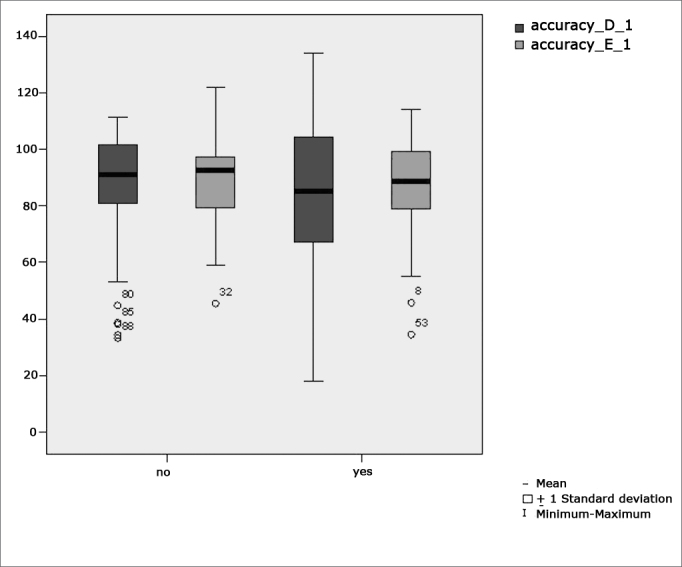
Box plot for right and left calibration eye-motor parameter (accuracy) per group. (Student”s t-test controlled by Lavene”s test for Equality of Variances between the means of parametric variables of interest).

Figure 3 features a box plot containing the statistical analysis for eye-motor calibration parameter accuracy.

Table 3 shows the results for Student”s t-test controlled by Levene”s test for Equality of Variances so as to check for possible differences between means of parametric variables for saccadic movement eye-motor parameters latency, velocity, and accuracy.

**Table 3:** Sample distribution in relation to eye-motor parameters of saccadic movements and school performance (n=88).

Variable	Learning impairment	n	Mean	Standard deviation	Significance (p)
	yes	43	215,23	98,26	
latency_D_2	no	45	176,46	58,14	0,123
	yes	43	101,74	29,19	
velocity_D_2	no	45	98,88	43,87	0,481
	yes	43	95,84	25,37	
accuracy_D_2	no	45	176,77	53,51	0,043*
	yes	43	103,26	27,67	
latency_E_2	no	45	97,91	28,58	0,274
	yes	43	108,55	33,14	
velocity_E_2	no	45	176,77	53,51	0,105
	yes	43	103,26	27,67	
accuracy_E_2	no	45	98,40	27,41	0,274

a≤0,05

**Figure 4:**
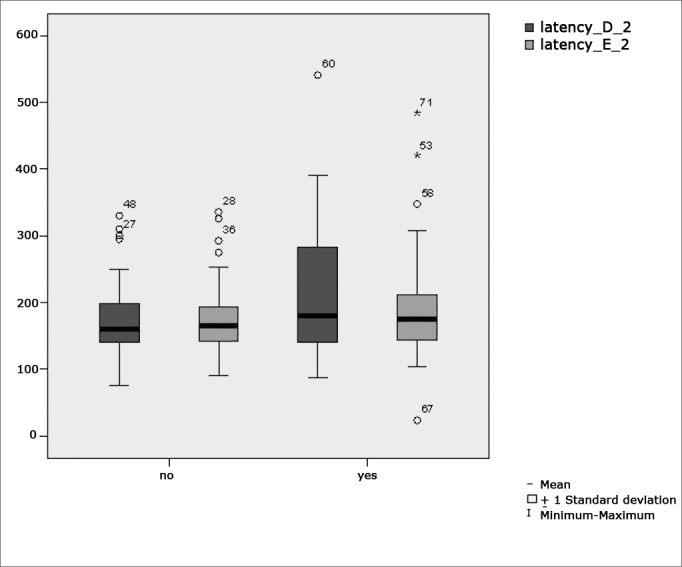
Box plot for right and left saccadic movement eye-motor parameter (latency) per group. (Student”s t-test controlled by Lavene”s test for Equality of Variances between the means of parametric variables of interest).

Figure 4 features a box plot containing the statistical analysis for saccadic movement eye-motor parameter latency.

**Figure 5:**
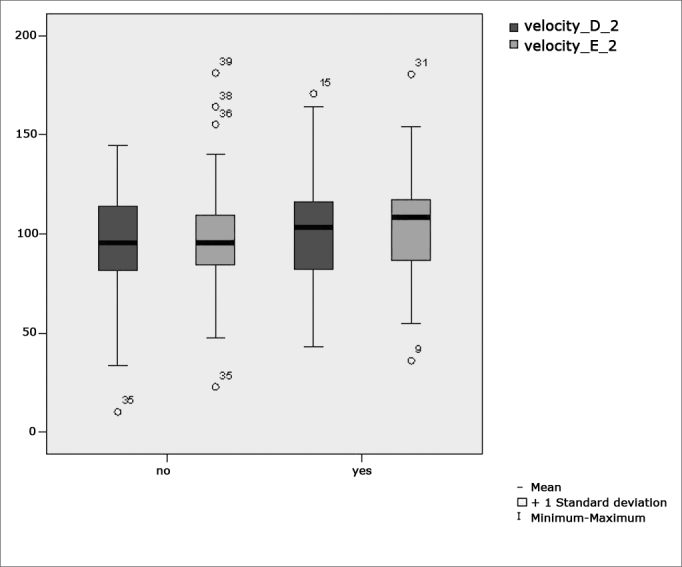
Box plot for right and left saccadic movement eye-motor parameter (velocity) per group. (Student”s t-test controlled by Lavene”s test for Equality of Variances between the means of parametric variables of interest).

Figure 5 features a box plot containing the statistical analysis for saccadic movement eye-motor parameter velocity.

**Figure 6:**
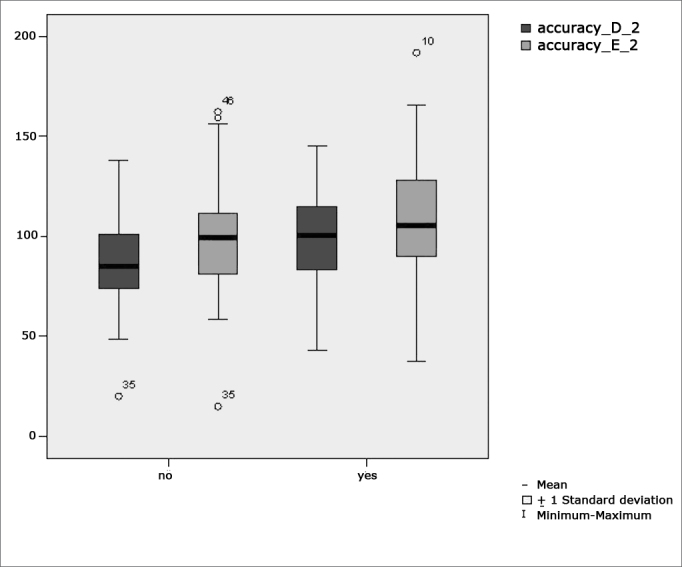
Box plot for right and left saccadic movement eye-motor parameter (accuracy) per group. (Student”s t-test controlled by Lavene”s test for Equality of Variances between the means of parametric variables of interest).

Figure 6 features a box plot containing the statistical analysis for saccadic movement eye-motor parameter accuracy.

Table 4 shows the results for Student”s t-test controlled by Levene”s test for Equality of Variances so as to check for possible differences between means of parametric variables for gains in pendulum tracking at 20Hz, 40Hz, and 80Hz.

**Table 4:** Sample in distribution in relation to pendulum tracking gains and school performance (n=88).

Variable	Learning impairment	n	Mean	Standard deviation	Significance (p)
	yes	43	0,91	0,36	
gain_20Hz	no	45	0,85	0,25	0,786
	yes	43	1,01	0,21	
gain_40Hz	no	45	0,98	0,22	0,695
	yes	43	0,86	0,23	
gain_80Hz	no	45	0,82	0,19	0,404

a≤0,05

**Figure 7:**
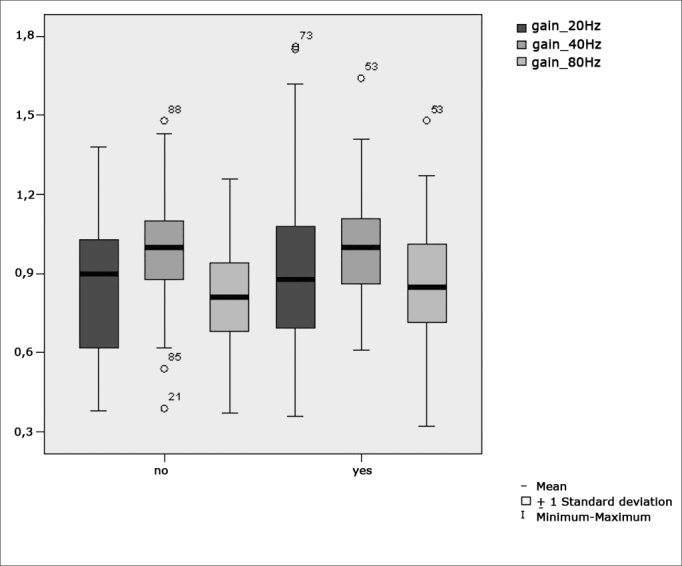
Box plot for parameter gain in pendulum tracking at 20Hz, 40Hz, and 80Hz per group. (Student”s t-test controlled by Lavene”s test for Equality of Variances between the means of parametric variables of interest).

Figure 7 features a box plot containing the statistical analysis for gains in pendulum tracking at 20Hz, 40Hz, and 80Hz

Table 5 shows the results for Student”s t-test controlled by Levene”s test for Equality of Variances so as to check for possible differences between means of parametric variables nystagmus directional preponderance (NDP) in the optokinetic test.

**Table 5:** Sample distribution in relation to nystagmus directional preponderance (NDP) in the optokinetic test and school performance (n=88).

Variable	Learning impairment	n	Mean	Standard deviation	Significance (p)
	yes	43	6,20	4,55	
OPTO_PDN	no	45	6,05	5,29	0,486
	yes	43	11,34	2,04	
VACL_D	no	45	10,55	2,37	0,075
	yes	43	10,92	2,01	
VACL_E	no	45	10,54	2,22	0,486

a≤0,05

**Figure 8:**
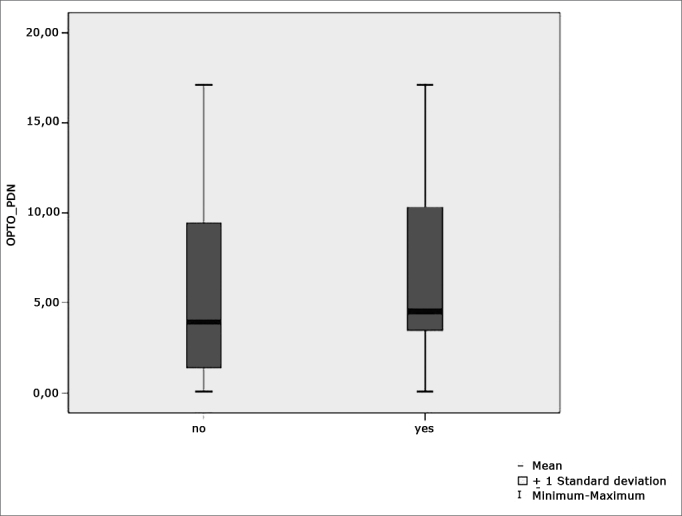
Box plot for nystagmus directional preponderance (NDP) in optokinetic tests per group. (Student”s t-test controlled by Lavene”s test for Equality of Variances between the means of parametric variables of interest).

Figure 8 features a box plot containing the statistical analysis for nystagmus directional preponderance (NDP) in the optokinetic test.

Table 6 shows the results for Student”s t-test controlled by Levene”s test for Equality of Variances so as to check for possible differences between means of nystagmus directional preponderance (NDP) in decreasing rotational pendulum test (DRPT) for lateral (NDP L), posterior (NDP P), and superior semicircular canals (NDP S).

**Table 6:** Sample distribution in relation to nystagmus directional preponderance (NDP) in decreasing rotational pendulum tests (DRPT) and school performance.

Variable	Learning impairment	n	Mean	Standard deviation	Significance (p)
	yes	43	10,42	7,63	
PDN_L	no	45	12,94	7,82	0,133
	yes	43	13,70	8,32	
PDN_P	no	45	13,01	7,55	0,726
	yes	43	13,31	8,74	
PDN_S	no	45	12,12	7,62	0,582

a≤0,05

**Figure 9:**
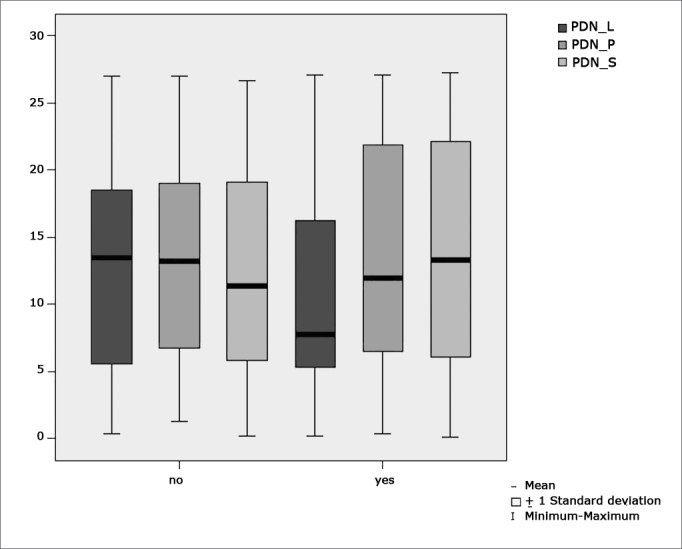
features a box plot containing the statistical analysis for nystagmus directional preponderance (NDP) in the decreasing rotational pendulum test (DRPT) for lateral (NDP L), posterior (NDP P), and superior semicircular canals (NDP S).

Figure 9 features a box plot containing the statistical analysis for nystagmus directional preponderance (NDP) in the decreasing rotational pendulum test (DRPT) for lateral (NDP L), posterior (NDP P), and superior semicircular canals (NDP S).

Table 7 shows the results for Student”s t-test controlled by Levene”s test for Equality of Variances so as to check for possible differences between means of parametric variables for nystagmus slow component angular velocity (SCAV) in the caloric test, at 42ºC and 18ºC for both ears.

**Table 7:** Sample distribution in relation to SCAV at 42ºC and 18ºC in both ears and school performance (n=88).

Variable	Learning impairment	n	Mean	Standard deviation	Significance (p)
	yes	43	23,34	13,30	
PC	no	45	16,76	9,65	0,012*
	yes	43	11,00	7,04	
D_42	no	45	8,81	3,04	0,478
	yes	43	10,99	5,44	
E_42	no	45	9,67	4,01	0,425
	yes	43	20,02	10,29	
D_18	no	45	14,69	7,36	0,012*
	yes	43	17,66	12,13	
E_18	no	45	12,43	6,56	0,031*

a≤0,05

**Figure 10:**
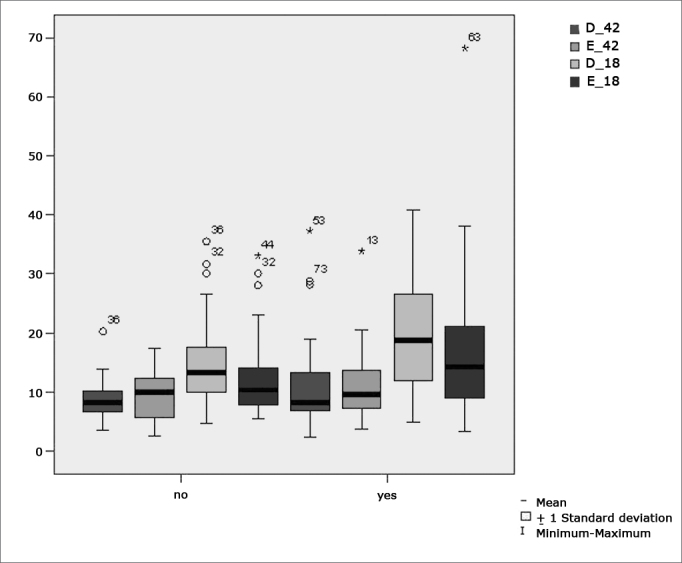
Box plot for right and left caloric tests (CT) at 18ºC and 42ºC per group. (Student”s t-test controlled by Lavene”s test for Equality of Variances between the means of parametric variables of interest).

Figure 10 features a box plot containing the statistical analysis for nystagmus slow component angular velocity (SCAV) in the caloric test at 42ºC and 18ºC for both ears.

Table 8 shows the distribution of the sample in relation to final diagnosis and school performance. Mann-Whitney”s test was used to assess differences and elicit the statistically significant relationship between the analyzed variables (p<0.001).

**Table 8:** Distribution of final diagnosis as a function of school performance (n=88).

Learning impairment	Final diagnosis					Total
	NVT	PIVS	SVPIB	SVPID	SVPIE	
No	33	2	6	3	1	45
	73,3%	4,4%	13,3%	6,7%	2,2%	100,0%
Yes	14	4	11	8	6	43
	32,6%	9,3%	25,6%	18,6%	14,0%	100,0%
Total	47	6	17	11	7	88
	53,4%	6,8%	19,3%	12,5%	8,0%	100,0%

p < 0,001 *

Legend:

NVT = normal vestibular test;

PIVS = peripheral irritative vestibular syndrome;

BPIVS = bilateral peripheral irritative vestibular syndrome;

RPIVS = right peripheral irritative vestibular syndrome;

LPIVS = left peripheral irritative vestibular syndrome.

## DISCUSSION

In our results we could verify the distribution of the sample in percent terms of school performance in relation to gender. Some similarity was observed (p= 0.121) between the analyzed variables, and no significant differences were seen between genders.

Forty-five (51.0%) of the 88 children included in the study did not report any difficulties at school, whereas 43 (49.0%) were underperforming.

Similar findings were reported by Farias et al.^2^ in a study that looked at 103 children with and without difficulties at school, 58.2% of whom did not report impairment and 41,7% did. Narciso et al.^7^ analyzed vestibular disorders in children and saw that 47% of them complained of low performance at school.

The remarks made by Schachter et al.^3^ and Sandler^4^ should be taken into account. In their studies they found that learning can be affected by genetic factors, peripheral sensorial conditions, and neurogenic diseases, among others. Within neurogenic disorders lie the conditions involving basic functional integration of the bodily scheme and or spatial orientation.

According to Guardiola et al.^5^, static and dynamic balance are important neurologic functions that enable children to adopt proper postures for learning. The authors claim that children with underdeveloped function in this area have an increased likelihood of experiencing learning difficulties.

Many are the contributions given by the studies done by Capovilla et al.^6^ and Campos et al.^8^ on the associations between the vestibular system and learning performance. Vestibular disorders in children may considerably impair their communication skills, psychological status, and school performance; the latter is a valuable indicative of labyrinthine disease.

According to Tuma et al.^10^, digital vectonystagmography is one of the most widely employed methods to assess vestibular function, as it grants additional diagnostic sensitivity by allowing the measurement of parameters connected to vestibular and eye-motor function and comparison between stimuli and responses, apart from identifying the direction in which such phenomena occur.

Our study found that the mean values associated with eye-motor parameters, both in the calibration of eye and saccadic movement, are within normal ranges for digital vectonystagmography according to Ganança et al.^24^ for latency, velocity, and accuracy. However, a statistically significant association was observed in the mean values for accuracy (p=0.043) in saccadic movement when comparing children underperforming at school to those doing well in class. Significance was found in only one parameter to the right side.

Marchesin et al.^13^ reported anomalies in voluntary saccade control in a wide variety of disorders such as dyslexia, learning disorders, and ADHD, thus supporting the compared data.

Our data sets seem to support the hypothesis postulated by Frank and Levinson^15^ in that vestibular disorders may interfere with sequential eye fixation required for reading.

These concerns have guided studies such as the one by Horak et al.^16^ in which the vestibular function of children with and without reading and writing disorders was analyzed. Altered vestibulo-ocular reflexes were found in 20% of the children underperforming at school. This adds to the findings reported by Hoyt^14^, as eye movement required for reading alternates between saccade movement and periods of fixation, thus requiring the integrity of the vestibular apparatus and of saccadic movements.

Our study showed that the mean values found in the caloric tests are within normal ranges for digital vectonystagmography as defined by Ganança et al.^24^ in hot thermal stimulation (42ºC), for both children underperforming at school and those doing well in class. However, values above the normal ranges defined by Ganança et al.^24^ were found in cold thermal stimulation (18ºC) of children underperforming at school, showing a statistically significant relationship between variables for both left and right stimulation (p= 0.031 and p=0.012 respectively).

Thermal stimulation is the most sensitive of the conventional vestibular tests. It allows the identification of vestibular disorders in patients without specific complaints and with normal results in other vestibular assessment steps.

The statement above was considered in the assumption that drove Ganança et al.^11^ to confirm that the caloric test is the vestibular test that presents the greatest number of alterations.

When looking at nystagmus labyrinthine or directional preponderance found in the caloric test, we observed a statistically significant association between variables when comparing children with and without learning impairment (p= 0.012). Our data sets show peripheral vestibular disorder combined with labyrinthine excitation, leading to vestibular hyperactivity.

Ganança^19^ studied 64 children with language disorders without complaints of dizziness and found high rates of vestibular disorders in peripheral topographic diagnosis.

When producing the diagnosis of the children enrolled in our study we found a high rate of normal vestibular test results (73.3%) among children doing well in class and lower rates (32.6%) among those with learning impairment. The identified vestibular disorders had both unilateral and bilateral irritative peripheral origins, and involved 67.4% of the children with learning impairment and 26.7% of the children performing well at school. There was a statistically significant association between variables (p= 0.001).

Our results agree with the finding reported by Ganança et al.^11^ when they studied 30 children complaining of learning impairment and found altered computerized nystagmography results in 20.0% of the children analyzed, all in peripheral topographic diagnosis.

Similar findings were reported by Franco and Caetanelli^20^. School-aged children underwent vestibular examination through vectonystagmography and 20.7% of them had both unilateral and bilateral peripheral vestibular disorders.

Sensorial stimuli are fundamental for the development of learning skills in children. When stimulated from an early age, children will develop god coordination, focus, creativity, and self-confidence, thus minimizing the chances of experiencing learning impairments.

Considering the relevance of the relationship between learning impairments and the vestibular system, further investigation must be conducted to confirm the findings reported in this study and to shed light on the ambiguities for which answers were not found.

In order for obscure aspects not to prevent proper intervention from being offered, more research and work on speech and hearing therapy is required.

## CONCLUSION

Our study showed that the mean accuracy values (in the assessment of saccadic movement) and the normal value thresholds in caloric tests specially under cold thermal stimulation (18ºC) were statistically significant in children complaining of learning impairment. A statistically significant relationship was also found for vestibular disorder and children affected by learning impairment. All found vestibular disorders are of a peripheral irritative origin.
